# IMU/UWB Fusion Method Using a Complementary Filter and a Kalman Filter for Hybrid Upper Limb Motion Estimation

**DOI:** 10.3390/s23156700

**Published:** 2023-07-26

**Authors:** Yutong Shi, Yongbo Zhang, Zhonghan Li, Shangwu Yuan, Shihao Zhu

**Affiliations:** 1 School of Aeronautic Science and Engineering, Beihang University, Beijing 100191, China; 17375384@buaa.edu.cn (Y.S.); zhonghan@buaa.edu.cn (Z.L.); yuanshangwu@buaa.edu.cn (S.Y.); 2205221@buaa.edu.cn (S.Z.); 2 Aircraft and Propulsion Laboratory, Ningbo Institute of Technology, Beihang University, Ningbo 315100, China

**Keywords:** motion estimation, inertial measurement unit (IMU), ultrawideband (UWB), Madgwick orientation filter, Kalman filter

## Abstract

Motion capture systems have enormously benefited the research into human–computer interaction in the aerospace field. Given the high cost and susceptibility to lighting conditions of optical motion capture systems, as well as considering the drift in IMU sensors, this paper utilizes a fusion approach with low-cost wearable sensors for hybrid upper limb motion tracking. We propose a novel algorithm that combines the fourth-order Runge–Kutta (RK4) Madgwick complementary orientation filter and the Kalman filter for motion estimation through the data fusion of an inertial measurement unit (IMU) and an ultrawideband (UWB). The Madgwick RK4 orientation filter is used to compensate gyroscope drift through the optimal fusion of a magnetic, angular rate, and gravity (MARG) system, without requiring knowledge of noise distribution for implementation. Then, considering the error distribution provided by the UWB system, we employ a Kalman filter to estimate and fuse the UWB measurements to further reduce the drift error. Adopting the cube distribution of four anchors, the drift-free position obtained by the UWB localization Kalman filter is used to fuse the position calculated by IMU. The proposed algorithm has been tested by various movements and has demonstrated an average decrease in the RMSE of 1.2 cm from the IMU method to IMU/UWB fusion method. The experimental results represent the high feasibility and stability of our proposed algorithm for accurately tracking the movements of human upper limbs.

## 1. Introduction

Motion capture (MoCap) systems provide technical support for the operation of space robots, which has enormously benefited the research into human–computer Interaction in the aerospace field. By capturing human upper limb movement and mapping it to space robot motion, space operators can remotely control space robot arms and allow them to perform complex and elaborate actions, such as grasping and handling. Thus, we need to accurately estimate the movement of human upper limbs [1,2,3]. Optical Mocap systems employ multiple cameras to capture the positions of reflective markers and have been widely used in human motion tracking. Although these systems share precise real-time tracking and strong anti-interference abilities, they are expensive and suffer from light and occlusion problems [4,5]. In order to flexibly track human movements, it is necessary to employ ubiquitous wearable sensors.

With the intensive study and rapid advancement of micro-electro-mechanical systems, wearable inertial measurement units (IMUs) have been extensively adopted for 3-D human motion tracking due to their low cost, portability, and high independence [6]. In many of the proposed IMU Mocap methods, accelerometers and gyroscopes are fused to estimate the body segment orientation [7,8], including the knee joint angle [9,10], arm adduction angle [11], and shoulder and elbow joint angles [12,13]. However, it has been illustrated that there is obvious drift error of the attitude angle calculated by the gyroscope, which contributes to the poor estimation accuracy and stability. Considering that the accelerometers can only compensate for the tilt angles relative to the direction of gravity, it is necessary to introduce magnetometers to provide compensation for the yaw angles, which is known as nine-axis IMU or the magnetic, angular rate, and gravity (MARG) system [14]. Data fusion of three inertial sensors can be achieved by adopting either a complementary filter [14,15,16,17] or a classic Kalman filter [18,19]. The Kalman filter has been widely used as an orientation filter for the majority of the proposed methods. Wu et al. [20,21,22,23] employed a particle filter and an unscented Kalman filter for information fusion and developed a micro-sensor Mocap system to achieve real-time tracking. Roetenberg et al. [24] designed a Kalman filter and improved the orientation estimation through the compensation of magnetic disturbances. Considering that the Kalman filter requires knowledge of noise distribution and is prone to computational load and parameter tuning issues, many researchers tend to use the complementary filter. Fourati et al. [25] proposed a complementary observer based on the quaternion method for motion tracking. However, only adopting inertial sensors fusion for motion estimation will still cause drift, so it is expected that a stable and high-precision technology will be introduced to fuse with IMU.

Considering the flexibility and high sampling rate of IMU, ultrawideband (UWB) technology is attractive for data fusion due to its low cost, portability, and low energy consumption [26,27], but it requires a clear line-of-sight (LOS) channel [28]. Compared with traditional UWB positioning methods consisting of maximum likelihood estimation (MLE), linearized least square estimation (LLSE), and weighted centroid estimation (WCE), the extended Kalman filter (EKF) performs with less computational time and less complexity [29]. Applying UWB and IMU fusion for human motion tracking can not only compensate for the low sampling rate of the UWB system, but reduce the drift of IMU as well. Depending on whether the fusion is based on raw time or location, data fusion methods are divided into tightly coupled [30] and loosely coupled [31,32] categories. Kok et al. [33] provided tightly coupled IMU and UWB fusion using an optimization method that showed good performance in terms of handling outliers, but suffered from clock skew challenges. Therefore, in order to facilitate the calculation, the loosely coupled method is often employed. Zihajehzadeh et al. [32,34,35] proposed a Kalman-filter-based IMU and UWB fusion method without a magnetometer and accurately captured lower body motion under magnetic disturbances. However, when extending the rotation matrices to the upper limb, their algorithm may suffer from the singularity problem. Zhang et al. [36] adopted a Mahony filter and quaternion for foot attitude estimation via IMU and UWB fusion, but the use of acceleration double integration to obtain the position would lead to huge cumulative errors.

In this paper, the human upper limb is taken as our research subject, and a novel IMU/UWB data fusion method is proposed for 3-D motion estimation by applying the Runge–Kutta Madgwick filter, the UWB localization Kalman filter, and the IMU/UWB Kalman filter. On the basis of the established kinematics model of the upper limbs, the quaternion method was employed to calculate the attitude angle to avoid the gimbal lock problem. Our proposed algorithm comprises the following two novel aspects: (1) we combined the Madgwick RK4 complementary orientation filter and Kalman filter for motion tracking, the Madgwick RK4 filter was employed to reduce gyroscope drift without leading to noise distribution, and the Kalman filter was implemented for UWB localization and fusion with known error distribution from the UWB system; and (2) the drift-free position obtained by the UWB localization system was used to fuse the position calculated by IMU for upper limb motion estimation, which enormously reduced the drift caused by the double integration of acceleration. The good experimental results represent the high feasibility and stability of our proposed algorithm.

The rest of the paper is structured as follows. In Section 2, the theoretical fusion method for 3-D upper limb tracking is described. The experimental setup and protocol are demonstrated in Section 3. Then, the experimental results are shown and analyzed in Section 4. Finally, conclusions are provided in Section 5.

## 2. Theoretical Method

**Figure 1:**
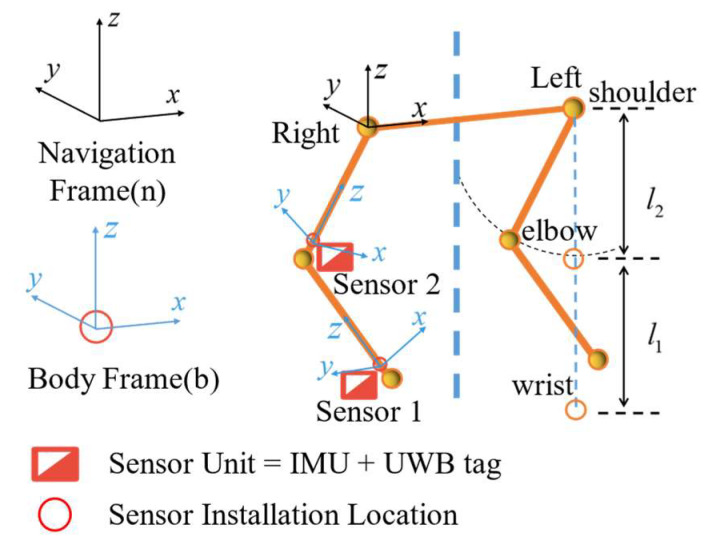
Upper limb kinematics model and sensor unit arrangement.

In this section, the proposed information fusion algorithm for human upper limb motion estimation is described. As shown in Figure 1, we first simplified the human upper limb as a kinematics model with three joints (shoulder, elbow, and wrist) and two segments (upper arm and forearm) [21]. The shoulder joint was set as a fixed point with three degrees of freedom (DoFs) and two DoFs for the elbow and wrist [37]. Taking the right arm as an example, two IMU sensors were arranged on the lateral side above the wrist and the elbow, respectively, as well as two UWB tags. The navigation coordinate frame (n) was set as the reference system, which was consistent with the coordinate system of UWB and MoCap. The body frame (b) was attached to each body segment where the sensors were located. It aligned with the n-frame initially. Neglecting the installation errors, the sensor frame (s) was attached to each IMU and aligned with the b-frame to track the movements of human upper limbs.

**Figure 2:**
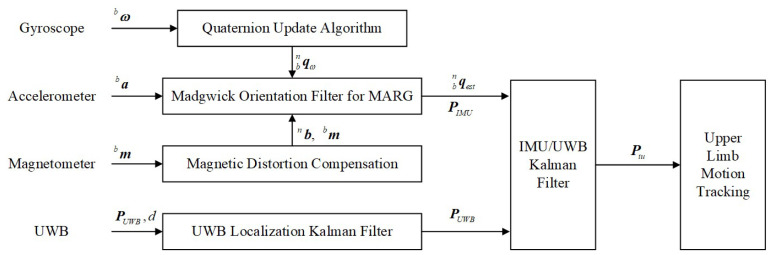
Framework of the proposed upper limb motion tracking algorithm.

The overall framework of the proposed algorithm consists of three parts, illustrated in Figure 2, including (1) Quaternion RK4 Based Madgwick Orientation Complementary Filter, (2) UWB localization Kalman filter, and (3) IMU/UWB Fusion Kalman filter. For the purpose of estimating the 3-D spatial trajectory of the movements of human upper limbs, we first employed the quaternion method to calculate the attitude angle using gyroscope measurements. However, the integration of gyroscope measurement errors contributed to an accumulating error in the quaternion algorithm [14]. Therefore, we considered adopting the Madgwick orientation filter to improve the motion estimation accuracy through the optimal fusion of the accelerometer, gyroscope, and magnetometer of the MARG system. Then, in order to further reduce the drift error, we proposed that the UWB localization system be combined with the IMU sensors. An extended Kalman filter was utilized to fuse IMU and UWB in order to perform stable and high-precision motion estimation of the human upper limbs.

### 2.1. Quaternion RK4 Based Madgwick Orientation Complementary Filter

#### 2.1.1. Fourth-Order Runge–Kutta-Based Quaternion Update Algorithm

Rotation transformation can be described as a vector rotating around a specified rotation axis with a certain angle in a coordinate system. The unit quaternion is defined as

(1) $$q=q_{1}+iq_{2}+jq_{3}+kq_{4}$$

For the same vector rn and rb, defined in the n-frame and b-frame, respectively, r′n and r′b are their extended forms, containing a 0 inserted as the real part [14]. qbn describes the rotation of the n-frame relative to the b-frame, so r′n can be expressed as

(2) r′n=qbn⊗r′b⊗q∗bn=qbn⊗M′q∗bnr′b=MqbnM′qbn∗r′b=q1−q2−q3−q4q2q1−q4q3q3q4q1−q2q4−q3q2q1q1q2q3q4−q2q1−q4q3−q3q4q1−q2−q4−q3q2q10xyz

(3) qbn⊗r′b=q1−q2−q3−q4q2q1−q4q3q3q4q1−q2q4−q3q2q10xyz

where $q^{*}$ is the conjugate of q; the symbol ⊗ denotes the quaternion product [21], as defined in (3); Mqbn is the left multiplication matrix of the quaternion qbn; and M′qbn∗ is the right multiplication matrix of the conjugate quaternion qbn∗. Furthermore, the rotation can be represented by a rotation matrix.

(4) rn=Cbnrb

where Cbn represents the rotation of the n-frame relative to the b-frame.

(5) Cbn=(q12+q22−q32−q42)2(q2q3−q1q4)2(q2q4+q1q3)2(q2q3+q1q4)(q12−q22+q32−q42)2(q3q4−q1q2)2(q2q4−q1q3)2(q3q4+q1q2)(q12−q22−q32+q42)

The quaternion update algorithm uses the angular velocity increment in the sample period measured by the IMU sensors to calculate the quaternion at each time in order to update the human motion data. The quaternion derivative is given by

(6) q˙bn=12qbn⊗ωb=12Mqbnωb=12M′ωbqbn    =12q1−q2−q3−q4q2q1−q4q3q3q4q1−q2q4−q3q2q10ωbxωbyωbz=120−ωbx−ωby−ωbzωbx0ωbz−ωbyωby−ωbz0ωbxωbzωby−ωbx0q1q2q3q4

where ωb=0ωbxωbyωbzT is the angular velocity measured by the gyroscope.

Given the initial value and the rotational angular velocity, the orientation at each time qbnω,t can be obtained by numerically integrating the quaternion derivative q˙bnω,t.

(7) qbnω,t=q^bnest,t−1+q˙bnω,tΔt=q^bnest,t−1+12Δtq^bnest,t−1⊗ωbt

where Δt is the sampling period, q^bnest,t−1 is the estimation of the quaternion at the previous time, and ωbt is the angular velocity at time t.

Although increasing the order of the above algorithm can improve the computational accuracy, the complexity is also increasing. The Runge–Kutta method is a high-precision single-step algorithm; the most classic and widely used is the fourth-order Runge–Kutta algorithm. It can perform iterative operations on definite solution problems with known initial values and equations without solving differential equations, which enormously reduces the computational complexity. In this paper, the calculation of human upper limb motion can be regarded as the initial value problem of a differential equation. The calculation formulas are as follows.

(8) Δq˙bn1=12q^bnest,t−1⊗ωbt−1Δq˙bn2=12q^bnest,t−1+Δq˙bn12⊗ωbt−1/2Δq˙bn3=12q^bnest,t−1+Δq˙bn22⊗ωbt−1/2Δq˙bn4=12q^bnest,t−1+Δq˙bn3⊗ωbtqbnω,t=q^bnest,t−1+16ΔtΔq˙bn1+2Δq˙bn2+2Δq˙bn3+Δq˙bn4

where ωbt−1/2 is the angular velocity at the intermediate time between t − 1 and t.

The quaternion fourth-order Runge–Kutta algorithm is designed to interpolate in the integration interval, and the slope is iteratively optimized at each step of the calculation to obtain an updated value.

#### 2.1.2. Madgwick RK4 Orientation Complementary Filter for MARG

By formulating an objective function, this filter fuses the data of the tri-axis accelerometer, gyroscope, and magnetometer of the MARG system, then iteratively optimizes it to calculate the orientation q^bn.

Let the predefined reference vector in the n-frame be d^n and the sensor measurement in the b-frame be s^b. The objective function is defined as

(9) minq^bn∈ℝ4 fq^bn,d^n,s^bfq^bn,d^n,s^b=q^bn∗⊗d^n⊗q^bn−s^bd^n=0dxdydzs^b=0sxsysz

By recording the initial quaternion q^bn0 and step size μ, the estimation of q^bnn+1 is obtained for n iterations, adopting the general gradient descent algorithm illustrated in (10). The symbol ∇ represents the gradient of the objective function, which can be computed by the objective function and its Jacobian, as shown in (11).

(10) q^bnk+1=q^bnk−μ∇fq^bnk,d^n,s^b∇fq^bnk,d^n,s^b, k=0,1,2⋯n

(11) ∇fq^bnk,d^n,s^b=JTq^bnk,d^nfq^bnk,d^n,s^b

The specific algorithm expression of the accelerometer is described in (12). Normalized gravity g^n and normalized accelerometer measurements a^b substituted d^n and s^b, respectively.

(12) g^n=0001a^b=0axayazfgq^bn,a^b=2q2q4−q1q3−ax2q1q2+q3q4−ay20.5−q22−q32−azJgq^bn=−2q32q4−2q12q22q22q12q42q30−4q2−4q30

The accelerometer compensated the tilt angle of motion estimation obtained by the gyroscope measurement, but the heading angle was not compensated, as this requires the introduction of a magnetometer. The magnetometer can be used to measure the strength and direction of the magnetic field and to determine the orientation of a device.

As for the magnetometer, the geomagnetic field b^n, as the substitution of d^n, can be decomposed into a horizontal axis and a vertical axis theoretically. Additionally, the normalized magnetometer measurement m^b is the substitution of s^b. The specific form can be written as

(13) b^n=00bybzm^b=0mxmymzfbq^bn,b^n,m^b=2byq1q4+q2q3+2bzq2q4−q1q3−mx2by0.5−q22−q42+2bzq1q2+q3q4−my2byq3q4−q1q2+2bz0.5−q22−q32−mzJbq^bn,b^n=2byq4−2bzq32byq3+2bzq42byq2−2bzq12byq1+2bzq22bzq2−4byq2+2bzq12bzq4−4byq4+2bzq3−2byq2−2byq1−4bzq22byq4−4bzq32byq3

However, the magnetometer may be disturbed by the bias of hard iron and soft iron, causing errors in the measurement direction of the earth’s magnetic field, so magnetic distortion compensation needs to be employed.

(14) h^nt=0hxhyhz=q^bnest,t−1⊗m^bt⊗q^bnest,t−1∗

(15) b^nt=00hx2+hy2hz

where h^nt is the normalized magnetometer measurement in the n-frame and b^nt is the compensated geomagnetic field at time t.

Combining the specific algorithms of the accelerometer and magnetometer, the formulas were developed as follows.

(16) fg,bq^bn,a^b,b^n,m^b=fgq^bn,a^bfbq^bn,b^n,m^bJg,bq^bn,b^n=JgTq^bnJbTq^bn,b^n

By substituting (16) into (11), the gradient of the combined objective function can be written as

(17) ∇f=Jg,bTq^bnest,t−1,b^nfg,bq^bnest,t−1,a^b,b^n,m^b

And the estimated quaternion qbn∇,t calculated at time t can be given by

(18) qbn∇,t=q^bnest,t−1−μt∇f∇f

Employing the Madgwick orientation filter, an estimated rotation qbnest,t was obtained by fusing qbnω,t and qbn∇,t.

(19) qbnest,t=γtqbn∇,t+1−γtqbnω,t, 0≤γt≤1

where $\gamma_{t}$ represents weight, ensuring that the weighted divergence of qbnω,t equals the weighted convergence of qbn∇,t.

According to [14], it is known that when noise augmentation of $\mu_{t}$ is assumed to be extremely high, (19) can be rewritten as

(20) qbnest,t=q^bnest,t−1+q˙bnest,tΔt=q^bnest,t−1+Δtq˙bnω,t−β∇f∇f

where β is the divergence rate of qbnω,t.

By substituting (8) into (20), it can be written in the fourth-order Runge–Kuta form as

(21) qbnest,t=q^bnest,t−1+Δt16Δq˙bn1+2Δq˙bn2+2Δq˙bn3+Δq˙bn4−β∇f∇f

The process of the quaternion RK4-based Madgwick orientation filter algorithm is summarized as follows in Algorithm 1.
**Algorithm 1:** Process of quaternion RK4-based Madgwick orientation complementary filter.**Initialization:**qbn0**Input:** ωbt,Δt,a^bt,m^bt,β**Output:** qbnest,t1: q˙bnω,t←0.5 q^bnest,t−1⊗ωbt2: qbnω,t←q^bnest,t−1+q˙bnω,tΔt3: Δq˙bn1,Δq˙bn2,Δq˙bn3,Δq˙bn4←q^bnest,t−1,ωbt−1,ωbt−1/2,ωbt4: qbnω,t←q^bnest,t−1+ΔtΔq˙bn1+2Δq˙bn2+2Δq˙bn3+Δq˙bn4/65: h^nt←q^bnest,t−1⊗m^bt⊗q^bnest,t−1∗; b^nt←h^nt6: fg,bq^bn,a^b,b^n,m^b←fgq^bn,a^bfbq^bn,b^n,m^bT7: Jg,bq^bn,b^n←JgTq^bnJbTq^bn,b^nT8: ∇f←Jg,bTq^bnest,t−1,b^nfg,bq^bnest,t−1,a^b,b^n,m^b9: qbnest,t←qbnω,t−Δtβ∇f∇f**Return:** qbnest,t

### 2.2. UWB Localization Kalman Filter

**Figure 3:**
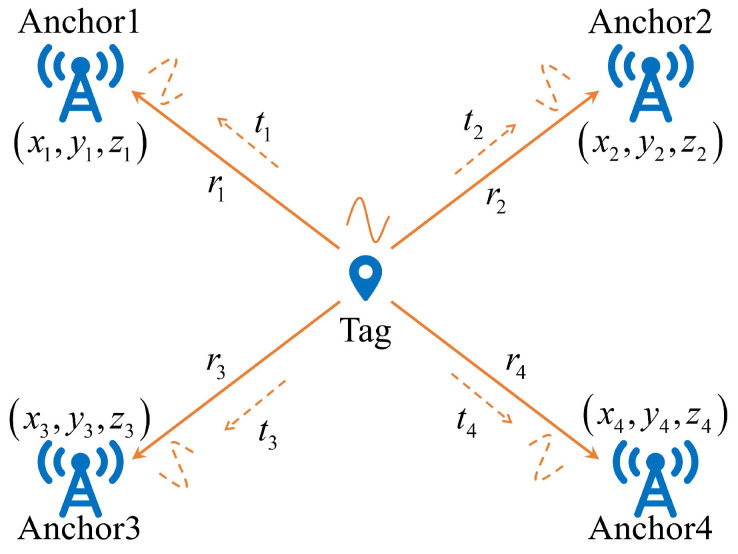
UWB positioning system.

The UWB positioning system contained a tag-anchor wireless communication channel, as shown in Figure 3. The calculation of the distances between each tag and anchor exploiting double-sided two-way ranging (DS-TWR) method and position estimation employed distances [28,29]. In addition to classical UWB positioning algorithms such as MLE, LLSE, and WCE, we used EKF for position estimation due to its lower complexity and shorter computational time [29].

It was defined that the UWB localization system was aligned with the n-frame. The system model adopted for UWB EKF algorithm can be expressed as

(22) $$\begin{array}{c} x_{u,t}^{}=x_{u,t-1}^{}+w_{u,t-1}^{}\\ y_{u,t}^{}=h_{u,t}\left(x_{u,t}^{}\right)+v_{u,t}^{} \end{array}$$

where the subscript u represents the UWB localization Kalman filter, and the state vector $x_{u,t}^{}=P_{UWB,t}={\left[\begin{array}{ccc} x_{t} & y_{t} & z_{t} \end{array}\right]}^{T}$ is a 3×1 vector where $P_{UWB,t}$ represents the 3-D position of UWB tags in the n-frame. The measurement vector $y_{u,t}^{}={\left[\begin{array}{cccc} d_{1,t} & d_{2,t} & d_{3,t} & d_{4,t} \end{array}\right]}^{T}$ denotes the distances between each anchor and tag. The expansion form of $h_{u,t}\left(x_{u,t}^{}\right)$ is expressed in (23). The process noise $w_{u,t-1}^{}$ and measurement noise $v_{u,t}^{}$ are zero mean additional Gaussian white noise with covariance matrices of $Q_{u,t-1}$ and $R_{u,t}$ respectively. These matrices were obtained from the UWB system, and distinguished the ranging accuracy of different anchors.

(23) $$h_{u,t}\left(x_{u,t}^{}\right)=\left[\begin{array}{c} \sqrt{{\left(x_{t}-x_{1}\right)}^{2}+{\left(y_{t}-y_{1}\right)}^{2}+{\left(z_{t}-z_{1}\right)}^{2}}\\ \sqrt{{\left(x_{t}-x_{2}\right)}^{2}+{\left(y_{t}-y_{2}\right)}^{2}+{\left(z_{t}-z_{2}\right)}^{2}}\\ \sqrt{{\left(x_{t}-x_{3}\right)}^{2}+{\left(y_{t}-y_{3}\right)}^{2}+{\left(z_{t}-z_{3}\right)}^{2}}\\ \sqrt{{\left(x_{t}-x_{4}\right)}^{2}+{\left(y_{t}-y_{4}\right)}^{2}+{\left(z_{t}-z_{4}\right)}^{2}} \end{array}\right]$$

where $x_{i},y_{i},z_{i},i=1,2,3,4$ represent the position of the i-th anchor.

Thus, the Jacobian matrix $H_{u,t}$ could be calculated as shown in (24). And for the sake of brevity, $D_{i,t},\;i=1,2,3,4$ is the corresponding row of $h_{u,t}\left(x_{u,t}^{}\right)$.

(24) $$H_{u,t}={\left.\frac{\partial h_{u,t}\left(x_{u,t}^{}\right)}{\partial x_{u,t}^{}}\right|}_{x_{u,t}^{}}=\left[\begin{array}{ccc} \frac{\left(x_{t}-x_{1}\right)}{D_{1,t}} & \frac{\left(y_{t}-y_{1}\right)}{D_{1,t}} & \frac{\left(z_{t}-z_{1}\right)}{D_{1,t}}\\ \frac{\left(x_{t}-x_{2}\right)}{D_{2,t}} & \frac{\left(y_{t}-y_{2}\right)}{D_{2,t}} & \frac{\left(z_{t}-z_{2}\right)}{D_{2,t}}\\ \frac{\left(x_{t}-x_{3}\right)}{D_{3,t}} & \frac{\left(y_{t}-y_{3}\right)}{D_{3,t}} & \frac{\left(z_{t}-z_{3}\right)}{D_{3,t}}\\ \frac{\left(x_{t}-x_{4}\right)}{D_{4,t}} & \frac{\left(y_{t}-y_{4}\right)}{D_{4,t}} & \frac{\left(z_{t}-z_{4}\right)}{D_{4,t}} \end{array}\right]$$

Assuming that $P_{u,0}$ is the initial state estimation covariance and is known as the prior, as well as the initial, state vector $x_{u,0}$, the posterior estimations $P_{u,t}$ and $x_{u,t}$ were recursively obtained by employing the prediction and update functions. It was defined that $P_{u,t|t-1}$ and $x_{u,t|t-1}$ were the prediction forms illustrated in (25) and could be calculated using the posterior estimations $P_{u,t-1}$ and $x_{u,t-1}$ at time t−1.

(25) $$\begin{array}{l} P_{u,t|t-1}=P_{u,t-1}+Q_{u,t-1}\\ x_{u,t|t-1}=x_{u,t-1} \end{array}$$

Then, the prediction and the measurement vector $y_{u,t}^{}$ were used to update the prior estimations, and the posterior estimations at time t could be expressed as

(26) $$\begin{array}{l} k_{u,t}=P_{u,t|t-1}H_{u,t}^{T}{\left[H_{u,t}P_{u,t|t-1}H_{u,t}^{T}+R_{u,t}\right]}^{-1}\\ x_{u,t}=x_{u,t|t-1}+k_{u,t}\left[y_{u,t}^{}-h_{u,t}\left(x_{u,t|t-1}\right)\right]\\ P_{u,t}=\left(I_{3\times 3}-k_{u,t}H_{u,t}\right)P_{u,t|t-1} \end{array}$$

where $x_{u,t}$ is the 3-D position of the UWB tag, and will be used as the measurement vector in the IMU/UWB Kalman filter.

### 2.3. IMU/UWB Fusion Kalman Filter

The innovative aspect of this IMU/UWB Kalman filter is that it uses the drift-free position calculated by the UWB system to compensate for the orientation and position estimated by the IMU system. The information obtained by the IMU system was employed as the state vector, and the position calculated by the UWB system was applied as the measurement vector. The system model can be expressed as

(27) $$\begin{array}{l} x_{iu,t}^{}=f_{iu,t-1}\left(x_{iu,t-1}^{},u_{iu,t-1}^{},w_{iu,t-1}^{}\right)\\ y_{iu,t}^{}=h_{iu,t}\left(x_{iu,t}^{},v_{iu,t}^{}\right) \end{array}$$

where the subscript iu represents the IMU/UWB Kalman filter. $x_{iu,t}$ is the state vector, $u_{iu,t-1}^{}$ is the input vector, $y_{iu,t}$ is the measurement vector, and $w_{iu,t-1}^{}$ and $v_{iu,t}^{}$ are the process noise and measurement noise, respectively.

The state vector xiu,t=PIMU,tqbnest,tT is a 7×1 vector where $P_{IMU,t}$ represents the 3-D position of human upper limb and qbnest,t is the orientation obtained from the Madgwick complementary filter. The state model consists of two parts, and the part qbnest,t can be expressed as

(28) qbnest,t=qbnest,t−1+Δtq˙bnest,t=qbnest,t−1+Δtq˙bnω,t−β∇f∇f=qbnest,t−1+Δt12qbnest,t−1⊗ωbt+ωbb,t−β∇f∇f

where ωbb,t is the bias of the gyroscope and $\frac{\nabla f}{\left\|\nabla f\right\|}$ is the input vector.

Supposing that $P_{IMU,0}$ is the initial position of the IMU sensors, then $P_{IMU,t}$ can be recursively calculated from a geometric perspective using the rotation quaternion qbnest,t or the rotation matrix Cbnt in (5), updated by qbnest,t. The position part of the state model is given by (29) and (30).

(29) PIMU,1u=Cbnu,1PIMU,0uTTPIMU,2u=Cbnu,2PIMU,0uTT=Cbnu,2Cbnu,1TPIMU,1uTTPIMU,3u=Cbnu,3PIMU,0uTT=Cbnu,3Cbnu,2TPIMU,2uTT            ⋯PIMU,tu=Cbnu,tCbnu,t−1TPIMU,t−1uTT

where $P_{IMU,t}^{u}$ and Cbnu,t represent the position and rotation matrix of the upper arm. The position of the forearm relies on the upper arm, and is given by

(30) PIMU,tf=PIMU,tu+Cbnf,tCbnf,t−1TPIMU,t−1f−PIMU,t−1uTT

where $P_{IMU,t}^{f}$ and Cbnf,t represent the position and rotation matrix of the forearm. When using the Kalman filter for the forearm, the input vector should contain the upper arm position.

The Jacobian matrices $F_{iu,t-1}$ and $L_{iu,t-1}$ were obtained.

(31) Fiu,t−1=∂fiu,t−1∂xiu,t−1xiu,t−1=CbntCbnt−1T03×404×3I4×4+12ΔtM′ωbt+ωbb,t

(32) Liu,t−1=∂fiu,t−1∂wiu,t−1xiu,t−1=03×412ΔtMqbnest,t−1

The measurement vector $y_{iu,t}^{}={\left[P_{UWB,t}\right]}^{T}$ is a 3×1 vector obtained from the UWB localization Kalman filter, representing the 3-D position of UWB tags in the n-frame. The measurement equation can be expressed as

(33) $$y_{iu,t}^{}=H_{iu,t}x_{iu,t}^{}+v_{iu,t}^{}$$

where $H_{iu,t}$ is the Jacobian matrix, expressed as

(34) $$H_{iu,t}=\left[\begin{array}{cc} I_{3\times 3} & 0_{3\times 4} \end{array}\right]$$

The covariance matrices $Q_{iu,t-1}$ and $R_{iu,t}$ of the process noise $w_{iu,t-1}^{}$ and the measurement noise $v_{iu,t}^{}$ were calculated as

(35) $$\begin{array}{cc} Q_{iu,t-1} & =E\left[w_{iu,t-1}^{}w_{iu,t-1}^{T}\right]=\sum_{G}=\sigma_{G}^{2}I_{4\times 4}\\ R_{iu,t} & =E\left[v_{iu,t}^{}v_{iu,t}^{T}\right]=\sum_{P,UWB}=\sigma_{P,UWB}^{2}I_{3\times 3} \end{array}$$

It was assumed that $P_{iu,0}$ and $x_{iu,0}$ were the initial state estimation covariance and state vector, respectively, and the prediction forms $P_{iu,t|t-1}$ and $x_{iu,t|t-1}$ could be recursively calculated using the following equations.

(36) $$\begin{array}{l} P_{iu,t|t-1}=F_{iu,t-1}P_{iu,t-1}F_{iu,t-1}^{T}+L_{iu,t-1}Q_{iu,t-1}L_{iu,t-1}^{T}\\ x_{iu,t|t-1}=f_{iu,t-1}\left(x_{iu,t-1}^{},u_{iu,t-1}^{},0\right) \end{array}$$

**Figure 4:**
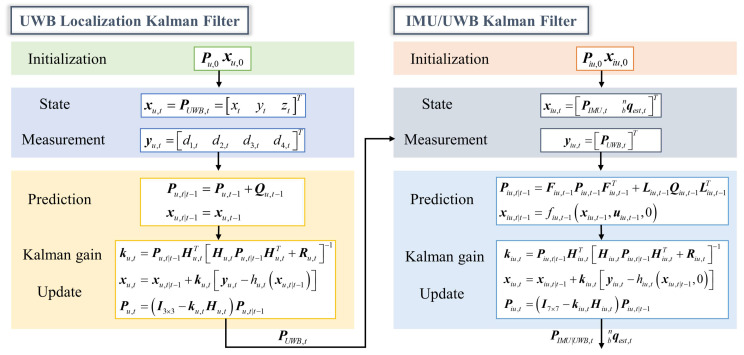
Flowchart of UWB localization and IMU/UWB Kalman filters.

Then, the prior estimations were updated, and the posterior estimations $P_{iu,t}$ and $x_{iu,t}$ were written as

(37) $$\begin{array}{l} k_{iu,t}=P_{iu,t|t-1}H_{iu,t}^{T}{\left[H_{iu,t}P_{iu,t|t-1}H_{iu,t}^{T}+R_{iu,t}\right]}^{-1}\\ x_{iu,t}=x_{iu,t|t-1}+k_{iu,t}\left[y_{iu,t}^{}-h_{iu,t}\left(x_{iu,t|t-1},0\right)\right]\\ P_{iu,t}=\left(I_{7\times 7}-k_{iu,t}H_{iu,t}\right)P_{iu,t|t-1} \end{array}$$

where $x_{iu,t}$ is the fused 3-D position in the IMU/UWB Kalman filter, which was used for 3-D motion reconstruction of human upper limbs. A detailed flowchart of the UWB localization and the IMU/UWB Kalman filters is shown in Figure 4. The parameters were set as follows. The initial state estimation covariance $P_{u,0}$ was set to $0.1\times I_{3\times 3}$. All elements of $P_{iu,0}$ were set to 0.1. $x_{u,0}$ and $x_{iu,0}$ were obtained from the UWB system and the optical MoCap system for each trial of movement, respectively.

## 3. Experimental Demonstration

### 3.1. Experimental Setup

To estimate the movement of human upper limbs, wearable 9-axis MPU9250 inertial sensor units (each sensor unit included a tri-axis accelerometer, a tri-axis gyroscope, and a tri-axis magnetometer) were adopted in our experiments. To avoid the extrusive effect of muscles of the human upper limbs, these two IMU sensors were placed on the lateral side, above the wrist and the elbow, respectively [11,21]. Each IMU sensor was connected to the computer via a serial port module for data transmission. The sample rate of the IMU sensors was set to 100 Hz, and the installation direction is shown in Figure 1.

**Figure 5:**
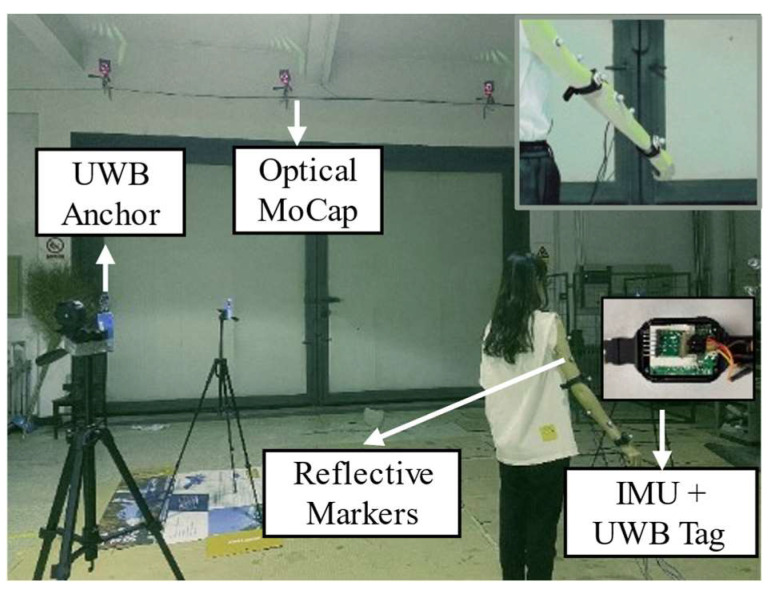
Setup of IMU sensors, UWB system, and optical MoCap system.

Additionally, a DW1000 UWB real-time localization system manufactured by Haoru Technology, Dalian, China, was employed to track the 3-D position with an update rate of about 10 Hz. The UWB communication technology was implemented between the anchors and tags through the DS-TWR method based on time-domain transmission of radio signals, and the anchors were also connected to the computer via a serial port module. It provided an accuracy of 10 cm for the X/Y axis and 30 cm for the Z axis. Two UWB tags were fixed on the bracelets with the IMU sensors to avoid relative movement, as shown in Figure 5. To ensure the positioning stability of UWB, four anchors were arranged in our laboratory in a cube distribution.

The Nokov optical MoCap system captures human motion through sixteen cameras evenly arranged in the laboratory, as illustrated in Figure 5, and is used as a reference system for algorithm verification and further comparison [34]. For each segment of the human upper limb, at least four reflective markers were attached to the skin surface and sensors, constituting an envelope rather than a cluster [9,11,38,39]. The optical MoCap system achieved sub-millimeter positioning accuracy and higher reliability.

### 3.2. Experimental Protocol

**Figure 6:**
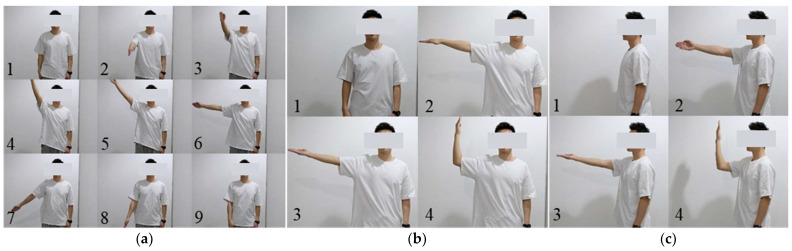
Tested movements. (**a**–**c**) represent motions 1–3, respectively.

Our experiments aimed to capture several common movements of the human upper limbs, including simple whole-arm movements and combined upper-arm and forearm movements. These common movements comprised motion 1: shoulder flexion/extension and abduction/adduction (the whole arm moving along a circular trajectory); motion 2: shoulder abduction/adduction, internal/external rotation, and elbow flexion/extension; and motion 3: shoulder flexion/extension, internal/external rotation, and elbow flexion/extension, as shown in Figure 6. In each trial, one type of movement was performed periodically, with a duration of about one minute.

**Figure 7:**
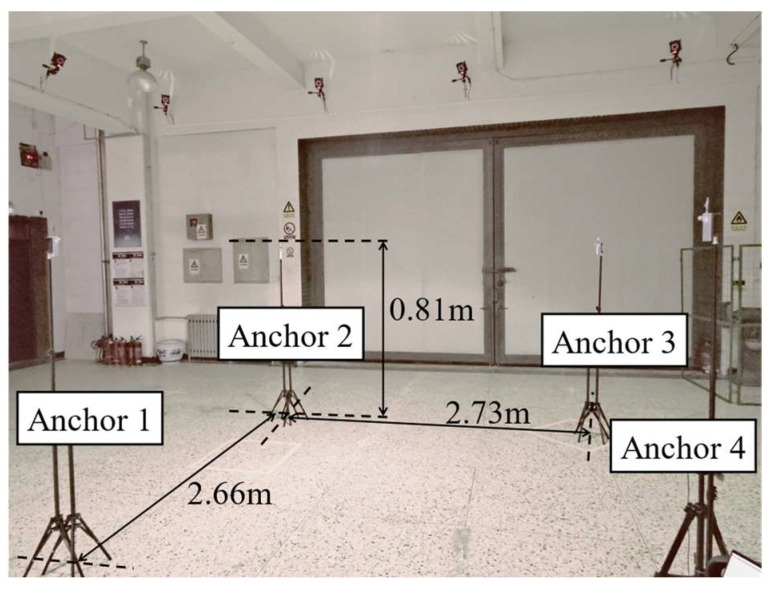
Cube distribution of four anchors of the UWB system.

As for the UWB system, four anchors arranged in conventional cube distribution were employed, as shown in Figure 7. The above movements were performed by one subject under each distribution throughout the experiment. The subject was a 24-year-old healthy female 165 cm in height and 40 kg in weight. It is important to mention that the optical MoCap system tracked the movement of the human upper limbs for each trial, which could be used to initialize the IMU sensors and obtain the positions of UWB anchors. Before each trial, the IMU sensors were calibrated, and a few seconds of stillness then enhanced the stability of the proposed algorithm.

## 4. Experimental Results and Discussion

### 4.1. Performance of Madgwick RK4 Orientation Filter

**Figure 8:**
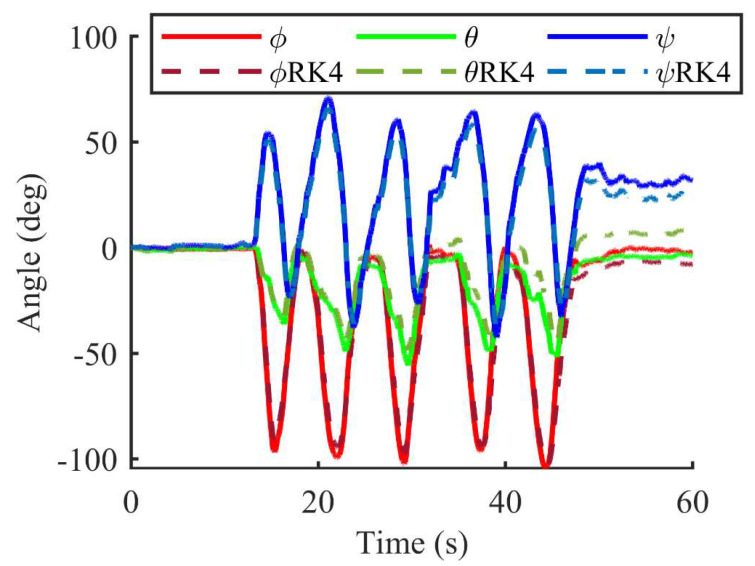
Euler angles of the forearm for motion 1 using the IMU system.

**Figure 9:**
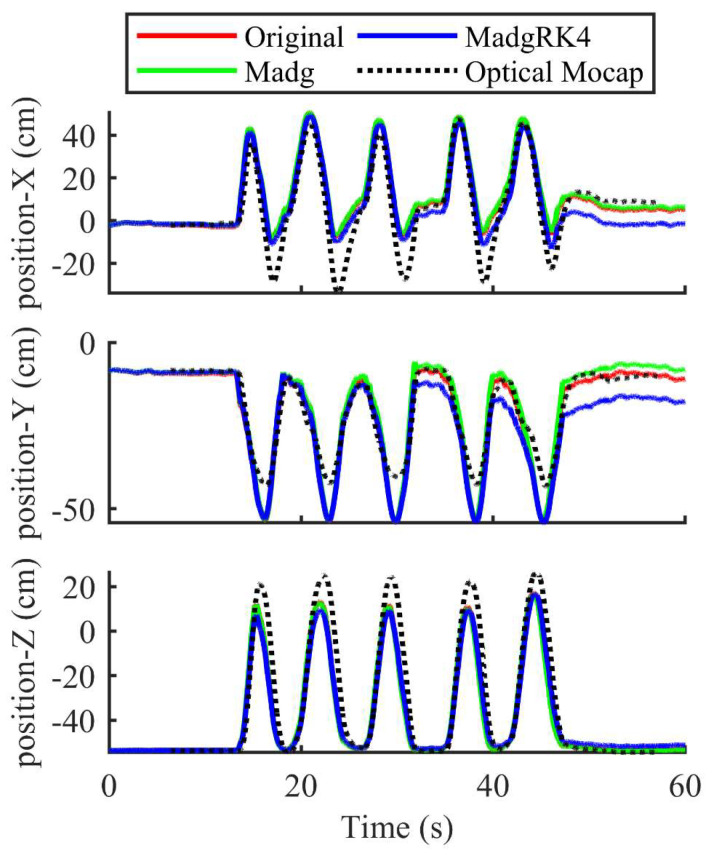
Positions of sensor unit 1 for motion 1.

The performance of the Madgwick orientation filter in terms of attitude angle and 3-D position estimation is shown in this section. Figure 8 shows the Euler angles of the wrist for motion 1, the whole arm moving along a circular trajectory, which is beneficial for us in verifying the tri-axis rotation (roll, pitch, and yaw) of the upper limbs. On the basis of the attitude angle obtained by the quaternion fourth-order Runge–Kutta algorithm and the original second-order update algorithm illustrated in (7), the Madgwick orientation filter was combined, as shown in (21) and (20), respectively, and the tri-axis positions of sensor unit 1 during the movement were calculated as shown in Figure 9. The solid lines represent the positions calculated using various methods from the IMU system, whereas the black dotted lines are the reference positions obtained by the optical Mocap system. As can be seen, the proposed methods were able to track the movements of the upper limbs in spite of the errors at the inflection points.

The position accuracies of the three kinds of movements are summarized in Table 1. It shows the 3-D position accuracy of forearm motion tracking by the fourth-order Runge–Kutta Madgwick method and the original second-order update algorithm compared with the optical Mocap system. According to Table 1, the root mean square error (RMSE) values of tri-axis motion tracking of Madgwick RK4 method varied from 6.17 cm to 12.76 cm, which shows the feasibility of precise motion tracking of the human upper limbs. In order to further reduce the error, it is necessary to utilize the UWB measurements.

### 4.2. Performance of UWB Localization Kalman Filter

As for the UWB localization Kalman filter, we utilized multiple sets of movements of the human upper limb to verify the accuracy of the UWB positioning system. It is important to mention that the installation direction of the IMU was always the same as that in Figure 1 during the experiment. And the subject stood at the edge of the UWB area to ensure that there was no occlusion between the tags and anchors during the movement of the upper limbs. Before the experiment, we first kept the tag still and verified the static positioning accuracy of UWB. Table 2 shows the static positioning variance of the four-anchor UWB system. The mean values of the tri-axis variances fell within 0.6 cm^2^, so it shared the good positioning robustness of the UWB system.

**Figure 10:**
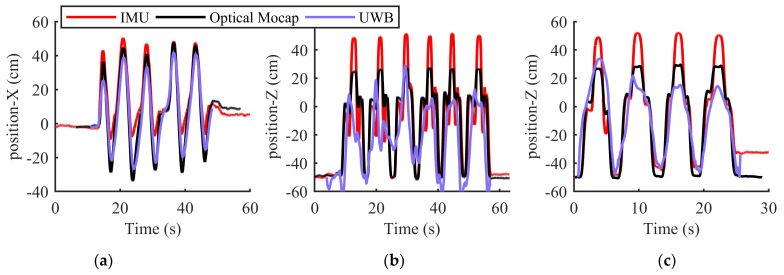
Comparison of the sensor unit 1 position of upper limb motion tracking for three kinds of movements by the IMU, UWB, and optical Mocap systems: (**a**–**c**) represent motion 1, motion 2, and motion 3, respectively.

Figure 10 shows a comparison of the position of upper limb motion tracking for three kinds of movements by the IMU, UWB, and optical Mocap systems. Motion 1, represented by Figure 10a, consisted of movement along a circular trajectory, which is consistent with of the motion in Figure 9. And motion 2 was a combined motion, represented by Figure 10b, comprising shoulder abduction/adduction, internal/external rotation, and elbow flexion/extension. Motion 3 was another combined motion, represented by Figure 10c and similar to motion 2, consisting of shoulder flexion/extension, internal/external rotation, and elbow flexion/extension. As can be seen in these figures, it is obvious that compared with the position calculated by the IMU sensors, the position calculated by the UWB system was closer to the reference value of the optical Mocap system at certain steps, especially the inflection points. Therefore, as for the measurement, the UWB system can be fused with IMU for upper limb motion estimation to reduce the error caused by the drift of the IMU sensors.

### 4.3. Performance of IMU/UWB Kalman Filter

The performance of the IMU/UWB Kalman filter in 3-D upper limb motion tracking is shown in this section. As mentioned previously, by adopting the cube distribution of four anchors, the measurement of UWB was fused with IMU for drift error reduction and better positioning accuracy in motion estimation. The position’s RMSE values are illustrated in Table 3, where motion 1, motion 2, and motion 3 are represented in Figure 10a–c, respectively. This shows a comparison of the 3-D position accuracy of various upper limb motion tracking methods, i.e., the IMU method, UWB method, and IMU/UWB fusion method with the optical Mocap system. Although the position accuracy of the UWB system may have sometimes fallen lower than that of the IMU, the adopted Kalman filter adaptively adjusted the weights between them based on the covariances. It can be observed that the tri-axis position RMSE calculated by the IMU/UWB Kalman filter was consistently less than that without UWB fusion. In our experiment, the proposed method was been extensively tested by various movements. For simple movements like motion 1, consisting of a circular trajectory, the tri-axis position RMSE values were all less than 10 cm. Compared with the IMU method, the relative errors calculated by the IMU/UWB fusion method were reduced by 40%, 3.6%, and 25.5% in the X-axis, Y-axis, and Z-axis, respectively. Complex combined movements such as motion 2 and 3 always consisted of multiple simple movements, such as shoulder flexion/extension, abduction/adduction, internal/external rotation, and elbow flexion/extension. Despite the fact that the tri-axis errors with combined motions are usually more significant than those with simple movements, the IMU/UWB fusion method still demonstrated better position accuracy than the IMU method, with a maximum RMSE of 12.2 cm. And our proposed methodology achieved an average decrease in the RMSE of 1.2 cm from the IMU method to the IMU/UWB fusion method. In comparison, by transferring the angle RMSE into position RMSE, the position RMSE values illustrated in Table 3 fell within the accuracy range of forearm motion without the constraints represented in [21].

**Figure 11:**
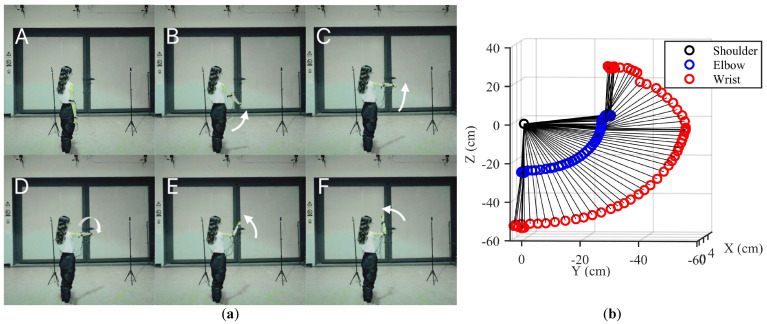
3-D spatial trajectory of sensor units 1 and 2 for motion 3. (**a**) Sequences of the real movement. (**b**) Motion reconstructed by the proposed algorithm.

Figure 11 shows a comparison of the 3-D spatial trajectory of upper limb motion reconstructed by the proposed algorithm and the real movement for motion 3. As can be seen in this figure, the proposed method was able to accurately track the movement of the human upper limb, and showed high feasibility and stability.

### 4.4. Power Consumption and Cost

According to the datasheet of the MPU9250 which was adopted in our experiment, the power consumption of the sensor depends on the selected operating mode and sensor configuration. In general, the device consumes between 8.3 μA and 3.9 mA. When the voltage is 3.3 V, the power consumption is as follows: in low-power mode, it ranges from 27.4 μW to 66.7 μW; in normal mode, with both the accelerometer and the gyroscope enabled, it varies from 31.5 μW to 764.7 μW; and in normal mode with all three sensors (accelerometer, gyroscope, and magnetometer) enabled, the power consumption ranges from 37.3 μW to 926.7 μW. The average price for a single MPU9250 chip is between USD 1 and 5.

As for the UWB system, according to the DW1000 datasheet, the typical power consumption values for the device in different modes are as follows: in idle mode, 24.75 mW; in receive mode, 56.84 mW; in transmit mode (at 6.8 Mbps data rate), around 270.76 mW; and in sleep mode, 20 nW. Typically, the cost of a single DW1000 module ranges from around USD 10 to 25.

However, the power consumption of an optical MoCap system can range from a few hundred watts to several kilowatts. This is because the cameras employed in this system require significant processing power and strict light conditions. And the cost of a basic system with a few cameras and basic software is around USD 1000. Therefore, compared with the optical MoCap system, the IMU/UWB system which we adopted exhibits extremely lower power consumption and is more economical and applicable.

## 5. Conclusions

In this paper, we proposed a novel method for hybrid upper limb motion tracking and 3-D positioning by fusing the IMU and UWB systems. We first simplified the human upper limb as a kinematics model and employed the quaternion method to calculate the attitude angle of each segment. To compensate for the accumulated error of gyroscope measurement, the fourth-order Runge–Kutta Madgwick orientation filter was adopted to improve the accuracy of 3-D motion tracking through the optimal fusion of the accelerometer, gyroscope, and magnetometer of the MARG system. The RMSE values varied from 6.17 cm to 12.76 cm, which shows feasibility for the precise motion tracking of human upper limbs. The error was mainly caused by drift of the IMU sensors. And it was inevitable that there would be a slight misalignment of coordinate frames at the initial moment.

In order to further reduce the drift error, we combined the UWB localization system with the IMU sensors. The static positioning variance of the four-anchor UWB system was tested, and the mean values of the tri-axis variances were within 0.6 cm^2^, so it shared good positioning robustness. By employing the UWB localization Kalman filter, the accuracy of UWB was verified by multiple sets of movements of the human upper limbs.

Adopting the four-anchor UWB system, we employed various movements to test the IMU/UWB Kalman filter. The experimental results represent that our proposed fusion algorithm achieved an average decrease in the RMSE of 1.2 cm from the IMU method to the IMU/UWB fusion method. With high feasibility and stability, our proposed algorithm was able to accurately track the movements of the human upper limbs.

In future work, we aim to study the effects of various distributions of multiple anchors on UWB localization accuracy, as well as to extend the proposed algorithm to the whole-body motion estimation.

## Figures and Tables

**Table 1:** The tri-axis position accuracy of forearm motion tracking by the Madgwick RK4 method and the original second-order update algorithm compared with the optical Mocap system.

	Position RMSE—Original Second -Order (cm)			Position RMSE—MadgwickRK4 (cm)		
	X	Y	Z	X	Y	Z
Motion 1	9.8361	4.9402	10.1491	8.6124	6.1745	9.3199
Motion 2	7.4554	7.5898	10.7254	6.6717	7.6876	10.5322
Motion 3	13.2050	11.1597	11.2611	12.7563	10.3026	11.9849

**Table 2:** Static positioning variance of the four-anchor UWB system.

Static Positioning Variance (cm^2^)			
	X	Y	Z
1	0.2970	0.4106	0.4080
2	0.2803	0.2255	0.7632
3	0.1668	0.3004	0.2671
4	0.1990	0.1649	0.2265
5	0.3813	0.4273	0.9591
mean	0.2649	0.3057	0.5248

**Table 3:** Comparison of the 3-D position accuracy of various upper limb motion tracking methods, i.e., the IMU method, UWB method, and IMU/UWB fusion method with the optical Mocap system.

	Position RMSE—IMU (cm)			Position RMSE—UWB (cm)			Position RMSE—IMU/UWB (cm)		
	X	Y	Z	X	Y	Z	X	Y	Z
Motion 1	9.1448	8.6423	8.5407	5.7678	4.3311	12.8657	5.4827	8.3291	6.3664
Motion 2	5.1256	8.5176	10.7520	11.5741	5.6851	15.9024	4.9606	7.8206	9.7357
Motion 3	12.7599	10.2743	11.9922	16.0117	12.5021	13.7803	12.2170	9.0845	11.1169

## Data Availability

Not applicable.
